# Potentiation by Protein Synthesis Inducers of Translational Readthrough of Pathogenic Premature Termination Codons in PTEN Isoforms

**DOI:** 10.3390/cancers16162836

**Published:** 2024-08-13

**Authors:** Leire Torices, Caroline E. Nunes-Xavier, Rafael Pulido

**Affiliations:** 1 Biobizkaia Health Research Institute, 48903 Barakaldo, Spain; ltorices@cicbiogune.es (L.T.); carolineelisabeth.nunes-xavier@bio-bizkaia.eus (C.E.N.-X.); 2 Institute for Cancer Research, Oslo University Hospital, 0424 Oslo, Norway; 3 Centro de Investigación Biomédica en Red de Enfermedades Raras, CIBERER, ISCIII, 28029 Madrid, Spain; 4 Ikerbasque, The Basque Foundation for Science, 48009 Bilbao, Spain

**Keywords:** translational readthrough, premature termination codon, genetic disease, precision therapy, PHTS

## Abstract

**Simple Summary:**

*PTEN* is an essential tumor suppressor gene whose alterations are causative of cancer and neurodevelopmental disorders, grouped as PTEN hamartoma tumor syndrome (PHTS). Here, we present translational readthrough as a potential therapeutic approach to restore the function of PTEN isoforms in patients harboring nonsense mutations at *PTEN* that cause aberrant PTEN biosynthesis. We have found that aminoglycosides and protein synthesis inducers reconstitute PTEN isoform expression and the function of *PTEN* variants containing nonsense mutations. Our results provide insights with which to optimize readthrough-based therapies for patients with cancer and PHTS.

**Abstract:**

The PTEN tumor suppressor is frequently targeted in tumors and patients with PTEN hamartoma tumor syndrome (PHTS) through nonsense mutations generating premature termination codons (PTC) that may cause the translation of truncated non-functional PTEN proteins. We have previously described a global analysis of the readthrough reconstitution of the protein translation and function of the human canonical PTEN isoform by aminoglycosides. Here, we report the efficient functional readthrough reconstitution of the PTEN translational isoform PTEN-L, which displays a minimal number of PTC in its specific N-terminal extension in association with disease. We illustrate the importance of the specific PTC and its nucleotide proximal sequence for optimal readthrough and show that the more frequent human PTEN PTC variants and their mouse PTEN PTC equivalents display similar patterns of readthrough efficiency. The heterogeneous readthrough response of the different PTEN PTC variants was independent of the length of the PTEN protein being reconstituted, and we found a correlation between the amount of PTEN protein being synthesized and the PTEN readthrough efficiency. Furthermore, combination of aminoglycosides and protein synthesis inducers increased the readthrough response of specific PTEN PTC. Our results provide insights with which to improve the functional reconstitution of human-disease-related PTC pathogenic variants from PTEN isoforms by increasing protein synthesis coupled to translational readthrough.

## 1. Introduction

PTEN protein is a central regulator of cellular growth and homeostasis, mainly via its phosphatase activity towards the 3′ position of the signaling lipid phosphatidylinositol (3,4,5)-trisphosphate (PIP3). PIP3 dephosphorylation by PTEN limits cell growth both during development and in the adult life, making PTEN an essential regulator of development and a strong tumor suppressor [1,2,3]. In addition, PTEN exerts regulation of cellular functions in the nucleus, including control of genome stability and gene transcription [4,5]. Decreased PTEN expression or alterations in PTEN functions are associated with human disease, including many types of cancers and several neurodevelopmental disorders [6,7,8]. PTEN is encoded by a unique gene, but different PTEN isoforms with N-terminal extensions of distinct length are produced via the alternative initiation of the translation of the PTEN mature mRNA, among which, PTEN-L/α constitutes the longest alternative isoform [9,10,11,12]. Diverse functions have been attributed to PTEN-L, although its relationship with pathogeny remains unclear.

*PTEN* gene mutations are associated with somatic and hereditary cancers, as well as with neurodevelopmental alterations, and heterozygous germline *PTEN* mutations define the PTEN hamartoma tumor syndrome (PHTS) disorder, a rare disease with a prevalence of about 1/200,000 [13,14]. An important number of *PTEN* mutations found in tumors and in patients, both in terms of gene distribution and frequency, are nonsense mutations distributed along most of the *PTEN* coding region and introducing premature termination codons (PTC) [15]. mRNA containing PTC can be degraded through nonsense-mediated mRNA decay (NMD) [16], and their potential translation produces truncated unstable or malfunctional PTEN proteins. Thus, approaches to reconstituting the expression and function of pathogenic PTEN targeted by PTC may be therapeutically beneficial in some cancers, as well as for specific patients with PHTS.

mRNA stabilization and reconstitution of full-length protein expression from PTC-targeted genes can be achieved by translational readthrough, which consists of the biosynthetic incorporation of an amino acid in the position of a termination codon by a near-cognate tRNA. Translational readthrough of PTC occurs naturally at a very low frequency during protein biosynthesis, but it can be increased in the presence of pharmacologic readthrough inducers, which constitutes the rationale of readthrough-based therapies for diseases associated with PTC-targeted genes [17,18,19]. The efficiency of reconstitution of full-length active proteins by readthrough inducers depends on the identity and the nucleotide context of the PTC, making specific experimental validations necessary [20,21]. Aminoglycoside antibiotics, such as G418/geneticin and gentamicin, are among the more extensively studied readthrough inducers, but due to their high toxicity, the search for efficient and non-toxic alternative readthrough inducers is under intense investigation [22,23]. In this regard, the protein synthesis process has been related to PTC readthrough efficiency, including a positive effect by the ribosome biogenesis and protein synthesis inducer Y-320 [24,25]. We have recently reported the global characterization of the translational readthrough of the complete PTEN PTC collection associated with disease (PTCome) and defined parameters that inform us of the suitability of the functional reconstitution by G418 of PTC-targeted PTEN [26]. Here, we have analyzed the PTC-readthrough of PTEN-L and evaluated the conditions that affect PTEN/PTEN-L readthrough. We have found that compounds that favor protein biosynthesis in cells increase PTEN/PTEN-L readthrough efficiency. These findings provide insights with which to improve therapeutic PTC-readthrough in patients with cancer and PHTS.

## 2. Materials and Methods

### 2.1. Analysis of PTC Distribution (PTCome)

The PTEN-L disease-associated PTCome was obtained from ClinVar [27] and HGMD Professional [28] databases, and it was visualized via Kernel density plot [15]. Human and mouse PTEN/PTEN-L nucleotide sequences were from entries NM_000314 and NM_008960, respectively. Human and mouse PTEN/PTEN-L amino acid sequences were from entries NP_000305 and NP_032986, respectively.

### 2.2. Cell Culture, Transfections, and Reagents

Simian kidney COS-7 and human embryonic kidney HEK293 cells were cultured as described [26]. Cells were transfected using GenJet reagent (SignaGen Laboratories) following the manufacturer’s instructions. Transfected cells were cultured for 24 h, followed by incubation in the absence or in the presence of readthrough or protein synthesis inducers, as indicated, and processed for analysis after additional culture for 24 h or 48 h, as previously described [15]. G418 (Geneticin, #345810; Merck Sigma Aldrich) was used at 200 μg/mL. Gentamicin (#345815; Merck Sigma Aldrich) was used at 800 μg/mL. PMA (4-β-phorbol-12-β-3- myristate-13-α-acetate, #P1585; Merck Sigma Aldrich) was used at 100 nM. Y-320 (#HY-15898; MedChemExpress) was used at 1 μM.

### 2.3. Plasmids and Mutagenesis

The plasmids pRK5 PTEN, pRK5 GST-PTEN (N-terminal tagging), pRK5 PTEN-GFP (C-terminal tagging), and pSG5 HA-AKT1 have been described in [29,30,31]. The plasmids pRK5 PTEN-L(Leu) (encoding PTEN-L starting with its starting Leu) and pRK5 PTEN-L(Met) (encoding PTEN-L starting with a starting Met) have been described in [32]. The plasmids pRK5 HA-PTEN and pRK5 HA-MMADHC (both N-terminal tagging) have been described in [33,34]. The plasmid pRK5 mPTEN (mouse sequence) was generated via substitution—using PCR and restriction enzymes—of the human PTEN sequence from pRK5 PTEN with the mouse PTEN sequence (NM_008960), obtained from the plasmid pCDNA3.1 mPTEN-V5, which was kindly provided by R. Parsons. The plasmid pRK5 HA-PTPN11 (N-terminal tagging) was generated via substitution—using PCR and restriction enzymes—of the PTEN sequence from pRK5 HA-PTEN with the PTPN11 human sequence, obtained from the plasmid pRK5 PTP-1D (PTPN11), which was kindly provided by A. Ullrich. Mutagenesis was performed via oligonucleotide site-directed PCR mutagenesis, as described [35], and mutations were confirmed via DNA sequencing. Nucleotide and amino acid numbering for human and mouse cDNAs used in the study accord with the entries indicated in Section 2.1.

### 2.4. Immunoblot and PTEN Functional Analysis

Cell lysates from transfected COS-7 or HEK293 cells were prepared, as described [26], using ice-cold M-PER^TM^ lysis buffer (ThermoFisher Scientific) supplemented with PhosSTOP phosphatase inhibitor and cOmplete protease inhibitor cocktails (Roche). A total of 50–100 μg of cell lysate proteins was resolved in 10% SDS-PAGE under reducing conditions and transferred to PVDF membranes. Experiments of PTEN functional activity in cells were performed co-transfecting the indicated PTEN variants and HA-AKT1, followed by monitoring of the phospho-AKT content. Immunoblots were performed with anti-PTEN 6H2.1 mAb (Merck Millipore) [36], anti-HA 12CA5 mAb (ThermoFisher Scientific), anti-phospho-Ser473-AKT, and anti-AKT (Cell Signaling Technologies), or anti-GAPDH (Santa Cruz Biotechnology, as described [26]. Protein bands were quantified using Image Studio^TM^ software with Odyssey^®^ CLx Imaging System (LI-COR Biosciences). For determination of PTC-readthrough efficiency, protein bands of full-length PTEN or PTEN-L generated by readthrough were quantified with respect to protein bands obtained with the wild-type proteins.

### 2.5. Statistical Analysis

For relevant comparative results, statistical significance was evaluated using the two-tailed Student’s *t* test in R. Significance was considered for *p*-values of *p* < 0.05, marked with one asterisk (*), or for *p*-values of *p* < 0.001, marked with two asterisks (**). Error bars represent mean ± standard deviation (SD) from at least two independent experiments.

## 3. Results

### 3.1. Distribution of Premature Termination Codons and Translational Readthrough of PTEN-L

**Figure 1:**
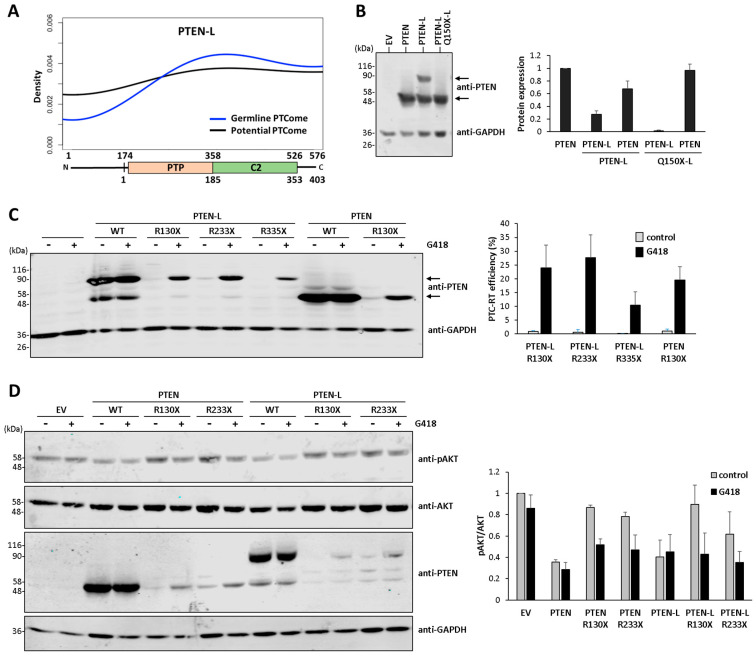
Distribution and translational readthrough of PTEN-L PTC associated with disease. (**A**) Kernel density plot of the distribution of the PTC on PTEN-L found in the germline of patients (germline PTCome). The potential PTCome includes all the PTC that can be generated via single-nucleotide substitutions, and it is shown as a control for sequence mutability. A schematic of PTEN-L domain composition is shown at the bottom (PTP, protein tyrosine phosphatase domain; C2, C2 domain). Numbers indicate PTEN-L amino acid numbering. (**B**) PTEN protein expression in the presence of PTEN-L PTC. COS-7 cells were transfected with empty vector (EV) or with plasmids encoding the indicated PTEN/PTEN-L [PTEN-L(Leu)] variants (WT, wild type), and protein expression was monitored via immunoblot using anti-PTEN 6H2.1 mAb, which recognizes the PTEN/PTEN-L common C-terminal region [36]. Immunoblot with anti-GAPDH antibody is shown as a loading control. The left panel shows a representative experiment, and arrows indicate the migration of PTEN-L and PTEN. The right panel shows the quantification of protein expression (relative to expression of PTEN ± SD) from at least two independent experiments. (**C**) Translational readthrough of PTC on PTEN-L. COS-7 cells were transfected with empty vector (EV) or with plasmids encoding the indicated PTEN or PTEN-L [PTEN-L(Met)] PTC variants (WT, wild type). Cells were kept untreated (−) or were treated (+) for 24 h with the readthrough inducer G418 (200 μg/mL), and readthrough was monitored via immunoblot with anti-PTEN 6H2.1 mAb. Immunoblot with anti-GAPDH antibody is shown as a loading control. The left panel shows a representative experiment, and arrows indicate the migration of PTEN-L and PTEN. The right panel shows quantification of readthrough, represented as PTC-readthrough (RT) efficiency ± SD (percentage of full-length PTEN or PTEN-L expression from PTC variants with respect to wild-type PTEN or PTEN-L) from at least two independent experiments. (**D**) Functional reconstitution of PTEN/PTEN-L through readthrough. COS-7 cells were co-transfected with pSG5 HA-AKT1 and empty vector (EV) or plasmids encoding the indicated PTEN/PTEN-L(Met) variants and processed for G418-induced readthrough, as in (**C**). The left panel shows a representative experiment. The right panel shows quantification of phospho-AKT (pAKT) content, represented as pAKT/AKT ± SD, relative to EV, from at least two independent experiments. The original Western blots are shown in Supplementary Material File S1.

We have previously described the collection of premature termination codons (PTC) present in the canonical coding region (403 amino acids) of the tumor suppressor *PTEN* gene in association with disease (PTEN disease PTCome), and we have made a global characterization of the translational readthrough of these PTC in response to aminoglycoside readthrough inducers [26]. Alternative translation initiation of PTEN mRNA generates PTEN long isoforms with non-structured N-terminal extensions followed by the PTEN PTP and C2 domains, of which PTEN-L/α is the longest isoform (576 amino acids in the human sequence [9,10,11]). Figure 1A shows the distribution of disease-associated PTC along the PTEN-L mRNA sequence, illustrating the very low frequency of PTC in the N-terminal PTEN-L region. The PTEN-L first amino acid is a Leu [PTEN-L(Leu)], and its translation is less efficient than the canonical PTEN, whose first amino acid is a Met (Figure 1B). As shown, a PTC (Q150X-L; amino acid numbering corresponds to PTEN-L) present in the PTEN-L N-terminal extension results in the lack of full-length PTEN-L expression and an increased translation of the full-length canonical PTEN protein (Figure 1B). These observations suggest that PTC in the N-terminal extension of PTEN-L are not detrimental for PTEN canonical function in terms of pathogeny. In this regard, a difference is observed between the species conservation of amino acid sequences when comparing canonical PTEN/PTEN-L specific sequences. Whereas human and mouse canonical PTEN proteins display the same length and only differ at one amino acid at the very C-terminus, human and mouse PTEN-L proteins differ in length (the human PTEN-L sequence contains an Ala stretch of 11 residues at its N-terminus, whereas the mouse PTEN-L sequence contains an Ala stretch of 7 residues at its N-terminus) and display 9 amino acids non-conserved at the PTEN-L N-terminal extension (Supplementary Figure S1; Supplementary Table S1). Thus, the interspecies conservation in the PTEN-L-specific amino acid sequence is relatively low when compared with the PTEN canonical sequence. Next, we tested the translational readthrough reconstitution of PTEN-L targeted by the more frequent PTEN PTC associated with human disease (R130X, R233X, and R335X; amino acid numbering corresponds to canonical PTEN) in comparison with PTEN. To facilitate visualization of PTEN-L, we used for these experiments a PTEN-L form starting with a Met at position +1 [PTEN-L(Met)] [32]. The aminoglycoside G418 efficiently induced the translation of full-length PTEN-L ectopically expressed in COS-7 cells, reaching readthrough levels comparable to those obtained with PTEN (Figure 1C). We also tested the functional reconstitution of PTEN/PTEN-L in cells upon readthrough-inducing conditions by analyzing phospho-AKT content in the presence of PTEN or PTEN-L PTC variants. As shown, the function of R130X and R233X PTEN/PTEN-L variants was reconstituted efficiently via G418 treatment (Figure 1D). Thus, biosynthesis and function of both PTEN/PTEN-L full-length proteins can be reconstituted upon translational readthrough induction.

### 3.2. Nucleotide Context and Readthrough Efficiency in Human and Mouse PTEN

**Figure 2:**
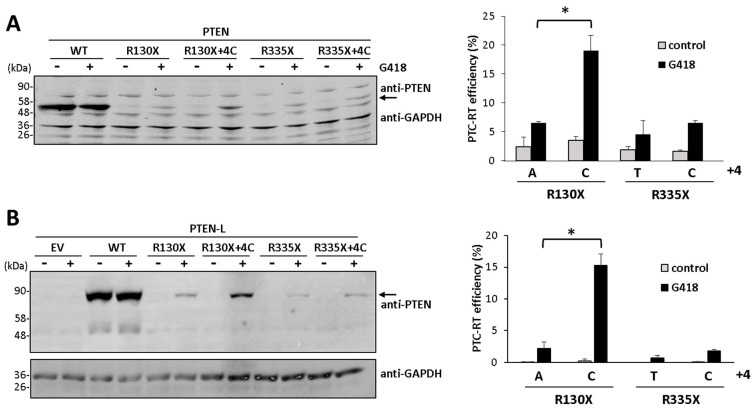
Nucleotide context and efficiency of PTC readthrough on PTEN-L and PTEN. (**A**,**B**) COS-7 cells were transfected with empty vector (EV) or with plasmids encoding PTEN (A) or PTEN-L(Met) (B) variants, as indicated (WT, wild type; +4C indicates a C in position +4, considering the first nucleotide of the PTC as +1), and processed for readthrough and immunoblot, as in Figure 1. The left panels show representative experiments and arrows indicate the migration of PTEN/PTEN-L. The right panels show quantification of PTC-readthrough (RT) efficiency ± SD, as in Figure 1 (+4 indicates the nucleotide at position +4). Statistical analysis was performed using an unpaired *t*-test (* = *p* < 0.05). The original Western blots are shown in Supplementary Material File S1.

**Figure 3:**
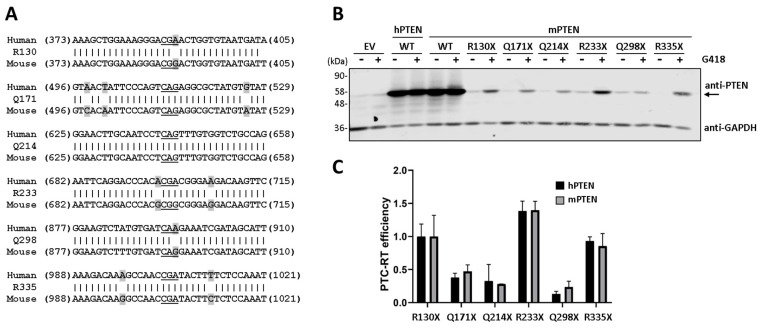
PTC readthrough of mouse PTEN. (**A**) Alignment of human and mouse PTEN nucleotide sequences flanking amino acids frequently targeted by PTC in human disease. The residue targeted by PTC is indicated, and the PTC is underlined. The numbers indicate nucleotide numbering according to entries NM_000314 and NM_008960. Differences in nucleotides are marked in grey. (**B**) Translational readthrough of PTC from mouse PTEN. COS-7 cells were transfected with empty vector (EV) or with plasmids encoding human PTEN (hPTEN) or mouse PTEN (mPTEN) variants, as indicated (WT, wild type), and processed for readthrough and immunoblot, as in Figure 1. The arrow indicates the migration of PTEN. (**C**) Comparative PTC-RT efficiency of PTC from human and mouse PTEN. The plot shows relative quantification of PTC-readthrough (RT) efficiency ± SD. The original Western blot is shown in Supplementary Material File S1.

Translational readthrough efficiency is influenced by the identity of the PTC as well as by the nucleotide context surrounding it [20,26]. This is illustrated for PTEN/PTEN-L in Figure 2, showing that a cytosine (C) nucleotide at position +4 (the first nucleotide in the PTC being +1) increases the readthrough efficiency of the R130X and R335X PTC. As mentioned above, human and mouse canonical PTEN sequences are highly conserved, rendering proteins almost identical and sharing an identity at the nucleotide level of about 96% in their coding regions. However, some nucleotide changes may affect the potential to generate disease-associated PTC (generally caused by one-nucleotide substitution) as well as the nucleotide context of the generated PTC (Figure 3A), which might affect the efficiency of PTC readthrough. Since mice models are suitable to test the translational readthrough of PTEN and its therapeutic potential in vivo, we tested the readthrough induction via the G418 of several human PTEN PTC in the context of the mouse PTEN nucleotide sequence, including those human PTEN PTC more frequently associated with disease. As shown, the readthrough efficiency of mouse PTEN was comparable to the readthrough efficiency of human PTEN in all cases tested (Figure 3B). Thus, G418-induced translational readthrough of the more prevalent PTEN PTC associated with disease in humans is efficiently achieved in mouse PTEN.

### 3.3. Protein Synthesis Rate Affects PTEN Readthrough Efficiency

**Figure 4:**
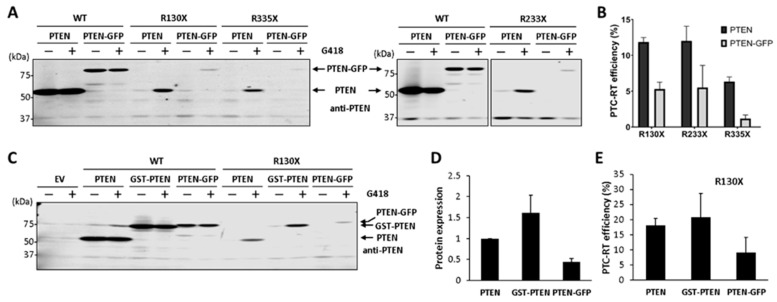
Readthrough efficiency of PTC variants from PTEN proteoforms synthesized at different levels. (**A**) COS-7 cells were transfected with plasmids encoding PTEN or PTEN-GFP variants, as indicated (WT, wild type), and processed for readthrough and immunoblot, as in Figure 1. (**B**) Quantification of PTC-readthrough (RT) efficiency of PTEN and PTEN-GFP variants, as in Figure 1. (**C**) COS-7 cells were transfected with empty vector (EV) or with plasmids encoding PTEN, GST-PTEN, or PTEN-GFP variants, as indicated (WT, wild type), and processed for readthrough and immunoblot, as in Figure 1. The arrows indicate the migration of PTEN, GST-PTEN, and PTEN-GFP. (**D**) Quantification of relative protein expression of PTEN, GST-PTEN, and PTEN-GFP. Relative protein expression ± SD is shown for wild-type (WT) proteins in the absence of G418 (−) relative to PTEN. (**E**) Quantification of PTC-readthrough (RT) efficiency of PTEN, GST-PTEN, and PTEN-GFP R130X variants, as in Figure 1. The original Western blots are shown in Supplementary Material File S1.

Comparative readthrough experiments using PTEN and PTEN-GFP (C-terminal tagging) showed a diminished induced readthrough efficiency for PTEN-GFP, which correlated with diminished protein synthesis in our ectopic expression system when compared with PTEN (Figure 4A,B). To analyze this effect in more detail, we tested, in parallel, the readthrough of GST-PTEN (N-terminal tagging), a fusion protein of similar size to PTEN-GFP that reaches expression levels similar to PTEN in our system. As shown, the relative expression levels of PTEN, GST-PTEN, and PTEN-GFP positively correlated with their readthrough efficiency (Figure 4C–E). Together, this suggests that a high protein synthesis rate increases the efficiency of PTEN translational readthrough.

**Figure 5:**
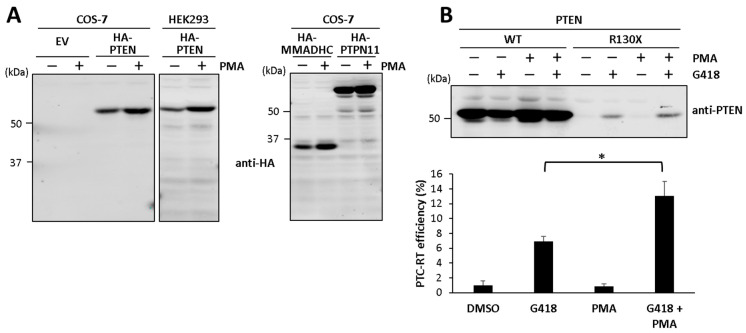
Effect of protein synthesis inducer PMA on the PTC-readthrough efficiency of PTEN. (**A**) COS-7 or HEK293 cells were transfected with empty vector (EV) or with plasmids encoding HA-PTEN, HA-MMADHC, or HA-PTPN11, as indicated. Cells were kept untreated (−) or were treated (+) for 24 h with PMA (100 nM), and protein expression was monitored via immunoblot with anti-HA 12CA5 mAb. (**B**) COS-7 cells were transfected with the indicated PTEN variants (WT, wild type), and incubated 24 h in the absence (−) or the presence of PMA (100 nM) or G418 (200 μg/μL), as indicated. Cells were processed for immunoblot, as in Figure 1. The upper panel shows a representative experiment, and the bottom panel shows quantification of PTC-readthrough (RT) efficiency ± SD, as in Figure 1. Statistical analysis was performed using an unpaired *t*-test (* = *p* < 0.05). The original Western blots are shown in Supplementary Material File S1.

Finally, we tested the G418-induced PTEN readthrough in the presence of PMA (4-β-phorbol-12-β-3- myristate-13-α-acetate), which is known to increase the protein synthesis rate in mammalian cells [37,38]. In our experiments, cell treatment with PMA augmented the ectopic expression of several non-related recombinant proteins of different sizes (HA-PTEN, HA-MMADHC, HA-PTPN11) upon transient transfection of both COS-7 and HEK293 cells, supporting the role of PMA as a general inducer of protein synthesis (Figure 5A). Cell treatment with PMA alone did not induce the translational readthrough of PTEN, but PMA increased the PTEN readthrough efficiency induced by G418 (Figure 5B). We conclude that a high protein synthesis rate of PTEN facilitates G418-induced PTEN PTC readthrough.

### 3.4. Potentiation of PTEN Readthrough by Y-320

**Figure 6:**
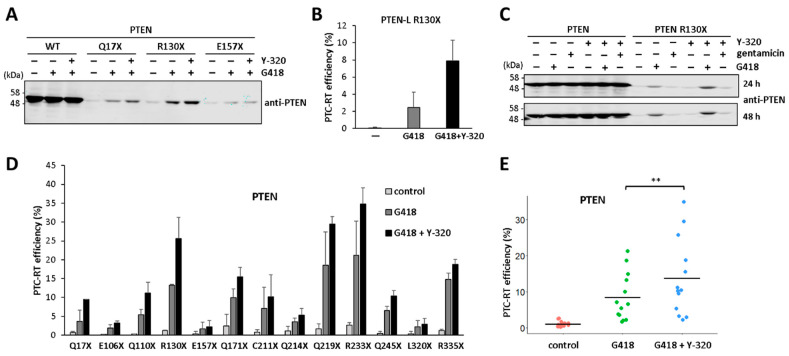
Effect of protein synthesis inducer Y-320 on the PTC-readthrough efficiency of PTEN. (**A**) COS-7 cells were transfected with the indicated PTEN variants (WT, wild-type) and incubated for 24 h in the absence (−) or the presence of Y-320 (1 μM) or G418 (200 μg/μL), as indicated. Cells were processed for immunoblot, as in Figure 1. (**B**) Quantification of PTC-readthrough (RT) efficiency of PTEN-L(Met) R130X in the absence (−) or in the presence of Y-320 (1 μM) or G418 (200 μg/μL), as indicated. PTC readthrough was monitored via immunoblot, as in Figure 1. (**C**) COS-7 cells were transfected with the indicated PTEN variants and incubated for 24 h or 48 h in the absence (−) or the presence of Y-320 (1 μM), gentamicin (800 μg/mL), or G418 (200 μg/μL), as indicated. Cells were processed for immunoblot, as in Figure 1. (**D**,**E**) Quantification of PTC-readthrough (RT) efficiency of selected PTEN PTC variants upon G418 or G418+Y-320 readthrough induction, as in panel A. In (**D**), readthrough efficiency is shown individually for each variant, as in Figure 1. In (**E**), readthrough efficiency is shown clustering the variants under the three different conditions. Statistical analysis was performed using a paired *t*-test (** = *p* < 0.001). The original Western blots are shown in Supplementary Material File S1.

The phenylpyrazoleanilide Y-320 compound has been described as an enhancer of protein synthesis and ribosome biogenesis, which increases the G418-induced translational readthrough of p53 [24]. Since we found a correlation between protein synthesis rate and the translational readthrough of PTEN facilitated by G418, we tested the readthrough effect of Y-320 in our PTEN readthrough experiments. As shown, Y-320, in combination with G418, increased the G418-induced readthrough of distinct PTC in PTEN/PTEN-L without manifesting any effect alone (Figure 6A,B). Y-320 also increased—albeit to a less extent—the PTEN R130X readthrough induced by gentamicin, another aminoglycoside that behaves as a weak readthrough inducer (Figure 6C). Next, we tested the effect of Y-320 on the readthrough of a selection of PTEN PTC associated with disease, and the results are shown in Figure 6D,E. An overall Y-320 potentiating readthrough effect on PTEN PTC is observed in the presence of G418, including the PTEN PTC more prevalent in human disease (R130X, R233X, and R335X). Thus, the Y-320 compound is a suitable potentiator of the translational readthrough of PTEN PTC induced by aminoglycosides.

## 4. Discussion

Translational readthrough constitutes a potential therapeutic option for human diseases caused by mutations generating PTC on specific genes. Since translational readthrough is highly dependent on intrinsic and extrinsic factors, dedicated in vitro studies that characterize the readthrough efficiency of readthrough inducers on the PTCome of relevant genes are necessary. Tumor suppressor genes are frequently targeted by PTC mutations in association with disease, and we have previously defined the readthrough efficiency in response to the aminoglycoside G418 of the PTCome of the PTEN tumor suppressor [26], which is mutated in somatic cancers and in the germline of patients with PHTS. Here, we have extended our observations to PTEN-L, the longest PTEN alternative translation isoform, and tested the PTEN PTC more commonly associated with disease for readthrough in the context of this isoform. Using our in vitro readthrough experimental model, we have found a similar biochemical and functional readthrough reconstitution of both PTEN/PTEN-L full-length proteins, despite their differences in amino acid composition and size. We show that disease-associated PTC target the N-terminal extension of PTEN-L with very low frequency, resembling what is observed for the PTEN C-terminal tail of canonical PTEN. This is indicative of regulatory rather than essential functions, associated with these two unstructured PTEN protein regions. In the case of PTC at the PTEN C-terminal tail, it seems that the resulting PTEN truncations do not fully compromise PTEN stability and function. In the case of PTC at the PTEN-L N-terminal extension, reinitiation of translation at the canonical PTEN Met1 neutralizes the protein truncating effect of the PTC. This should be taken into consideration when evaluating the extent of the potential pathogenicity associated with PTC mutations in the regions of the PTEN gene encoding these unstructured N- and C-terminal PTEN elements. Several specific functions have been attributed to PTEN-L [39,40,41]. The lack of disease-associated PTC in its N-terminal extension suggests that PTEN-L functions related with PIP3 dephosphorylation can be compensated by canonical PTEN.

We illustrate how the identity of the PTC and its nucleotide context affect the readthrough efficiency of human and mouse PTEN, which is important when evaluating the potential efficacy of readthrough therapies in patients with PHTS, as well as in choosing mouse models for in vivo PTEN readthrough studies. Importantly, the more frequent human PTEN PTC associated with disease (R130X, R233X, and R335X) display efficient G418-induced readthrough in the background of both human and mouse DNA sequences. It is worth mentioning that the nucleotide codons for mouse R130 and R233 PTEN residues do not generate a PTC upon a single-nucleotide substitution (Figure 2B), as happens in the case of human PTEN. In addition, we propose the protein synthesis rate as a factor that positively regulates G418-induced PTEN readthrough. This is relevant in the context of PTEN expression regulation in patients with PHTS during development or in response to nutritional and environmental conditions. We speculate that increasing the translation rate of PTEN might be beneficial in the case of potential readthrough-based therapies. In this regard, a regulatory loop of PTEN translation mediated by mTOR and driven by the activity of the PI3K pathway under physiologic or oncogenic conditions has been described [42].

Toxicity of G418 is not compatible with its therapeutic use, whereas other aminoglycosides, such as gentamicin, although much weaker readthrough inducers, have been used as systemic or topical treatments in pilot therapeutic studies [43,44,45]. Gentamicin also has undesirable nephrotoxic and ototoxic side-effects [46], making the search and validation of alternative readthrough-inducing compounds necessary [22]. In this regard, we report a positive effect of the small molecule Y-320 on the G418- and gentamicin-induced PTEN readthrough. This is in line with the findings from Hosseini-Farahabadi et al., who reported the synergistic induction of p53 readthrough by Y-320 in association with the stimulation of ribosome biogenesis and protein synthesis [24]. In addition, Heldin et al. have recently identified novel compounds that cooperate with G418 to induce p53 and PTEN readthrough [47]. Together, these findings reinforce the potential of PTEN readthrough-based therapies and provide novel perspectives from which to use and validate non-toxic agents as efficient drugs in specific groups of patients with cancer and PHTS.

## 5. Conclusions

Biochemical and functional reconstitution of specific PTEN/PTEN-L PTC variants harboring nonsense mutations is achieved upon aminoglycoside-induced translational readthrough. Stimulation of protein synthesis increased the G418-induced readthrough of PTEN. Combinatorial use of non-toxic readthrough-inducing compounds and protein synthesis stimulation could have therapeutic potential for patients with cancer and PHTS.

## Data Availability

All relevant data are included within the paper and its Supplementary Materials.

## Supplementary Materials

The following supporting information can be downloaded at https://www.mdpi.com/article/10.3390/cancers16162836/s1, Figure S1: CLUSTAL alignment of human and mouse PTEN-L amino acid sequences. Table S1: Human and mouse PTEN-L and PTEN amino acid differences. Files S1: Original Western Blot images.
