# Supplementary material for: Potentiation by Protein Synthesis Inducers of Translational Readthrough of Pathogenic Premature Termination Codons in PTEN Isoforms
Source: Cancers (Basel). 2024 Aug 13;16(16):2836. doi: 10.3390/cancers16162836 (PMC11352852; doi:10.3390/cancers16162836)
Supplement: Supplementary file 1 [file cancers-16-02836-s001.zip › cancers-3095202-supplementary.pdf]

Figure S1: Alignment of human PTEN-L (hPTEN-L, NP_ 000305) and mouse PTEN-L (mPTEN-L, NP_032986) amino acid sequences.

```
                                    29       38               54

hPTEN-L    LERGGEAAAAAAAAAAAPGRGSESPVTISRAGNAGELVSPLLLPPTRRRRRRHIQGPGPV     60
mPTEN-L    LERGGEAA----AAAAAPGRGSESPVTIARAGNAGELLSPLLLPPTRRRRRRHVQGPGPV     56
           ********    ****************:********:***************:******
                                    25       34               50

            62         72         85  89       97
hPTEN-L    LNLPSAAAAPPVARAPEAAGGGSRSEDYSSSPHSAAAAARPLAAEEKQAQSLQPSSSRRS     120
mPTEN-L    LSLPSAAAAPPLARAPEAAGGGSRCEDYPSSPHSAASAARPLAAEEKQAQSLQPSSSRRS     116
           *.*********:************.*** *******:***********************
            58         68         81  85       93

                 129
hPTEN-L    SHYPAAVQSQAAAERGASATAKSRAISILQKKPRHQQLLPSLSSFFFSHRLPDMTAIIKE     180
mPTEN-L    SHYPAAVQGQAAAERGASATAKSRAISILQKKPRHQQLLPSLSSFFFSHRLPDMTAIIKE     176
           ********.***************************************************
                 125

hPTEN-L    IVSRNKRRYQEDGFDLDLTYIYPNIIAMGFPAERLEGVYRNNIDDVVRFLDSKHKNHYKI     240
mPTEN-L   IVSRNKRRYQEDGFDLDLTYIYPNIIAMGFPAERLEGVYRNNIDDVVRFLDSKHKNHYKI      236
           ************************************************************

hPTEN-L    YNLCAERHYDTAKFNCRVAQYPFEDHNPPQLELIKPFCEDLDQWLSEDDNHVAAIHCKAG     300
mPTEN-L    YNLCAERHYDTAKFNCRVAQYPFEDHNPPQLELIKPFCEDLDQWLSEDDNHVAAIHCKAG     296
           ************************************************************

hPTEN-L    KGRTGVMICAYLLHRGKFLKAQEALDFYGEVRTRDKKGVTIPSQRRYVYYYSYLLKNHLD     360
mPTEN-L    KGRTGVMICAYLLHRGKFLKAQEALDFYGEVRTRDKKGVTIPSQRRYVYYYSYLLKNHLD     356
           ************************************************************

hPTEN-L    YRPVALLFHKMMFETIPMFSGGTCNPQFVVCQLKVKIYSSNSGPTRREDKFMYFEFPQPL     420
mPTEN-L    YRPVALLFHKMMFETIPMFSGGTCNPQFVVCQLKVKIYSSNSGPTRREDKFMYFEFPQPL     416
           ************************************************************

hPTEN-L    PVCGDIKVEFFHKQNKMLKKDKMFHFWVNTFFIPGPEETSEKVENGSLCDQEIDSICSIE     480
mPTEN-L    PVCGDIKVEFFHKQNKMLKKDKMFHFWVNTFFIPGPEETSEKVENGSLCDQEIDSICSIE     476
           ************************************************************

hPTEN-L    RADNDKEYLVLTLTKNDLDKANKDKANRYFSPNFKVKLYFTKTVEEPSNPEASSSTSVTP     540
mPTEN-L    RADNDKEYLVLTLTKNDLDKANKDKANRYFSPNFKVKLYFTKTVEEPSNPEASSSTSVTP     536
           ************************************************************

                                             571
hPTEN-L    DVSDNEPDHYRYSDTTDSDPENEPFDEDQHTQITKV    576
mPTEN-L    DVSDNEPDHYRYSDTTDSDPENEPFDEDQHSQITKV    572
           ******************************:*****
                                         567
```

Alignment was performed using CLUSTAL (Madeira F et al., Nucleic Acids Research, 10 Apr 2024, :gkae241. doi.org/10.1093/nar/gkae241. PMID: 38597606). Differences in amino acids are marked in cyan, with indications of the amino acid numbers. The first Leu (L) from PTEN-L and the first Met (M) from PTEN are marked in yellow

Table S1: Differences in amino acid sequences from human and mouse PTEN-L and PTEN

Human PTEN-L N-terminal sequence (173 amino acids	Mouse PTEN-L N-terminal sequence (169 amino acids)
S29-L	A25-L
V38-L	L34-L
I54-L	V50-L
N62-L	S58-L
V72-L	L68-L
S85-L	C81-L
S89-L	P85-L
A97-L	S93-L
S129-L	G125-L
Amino acid substitution rate = 0.052	
Human PTEN sequence (403 amino acids)	Mouse PTEN sequence (403 amino acids)
T398	S398
Amino acid substitution rate = 0.002	

Amino acid nomenclature numbering in PTEN-L is according to Pulido et al, Sci Signal. 2014 Jul 1;7(332):pe15 .
